# The minimal effective volume (MEAV 95) for interscalene brachial plexus block for surgical anesthesia under sedation: A prospective observational dose finding study

**DOI:** 10.1080/24740527.2017.1304805

**Published:** 2017-06-26

**Authors:** Stephen Choi, Jane J. Wang, Imad T. Awad, Paul McHardy, Ben Safa, Colin J. McCartney

**Affiliations:** a Department of Anesthesia, Sunnybrook Health Sciences Centre, Toronto, Ontario, Canada; b Department of Anesthesiology, The Ottawa Hospital, Ottawa, Ontario, Canada

**Keywords:** Interscalene block, Phrenic nerve, sedation

## Abstract

**Background**: Interscalene brachial plexus (ISB) block is routinely used to provide anesthesia and analgesia for shoulder surgery. Traditional local anesthetic volumes for ISB result in near universal ipsilateral phrenic nerve paresis potentially including oxygenation and ventilation.

**Aims**: The purpose of this study was to determine the lowest minimal effective anesthetic volume in 95% of patients (MEAV 95) of ropivacaine 0.75% for ISB that provides surgical anesthesia for arthroscopic shoulder surgery.

**Methods**: Prospective observational cohort study in patients undergoing arthroscopic shoulder surgery under ISB (C6 level) with sedation. The dose finding protocol used the Narayana rule for up/down sequential allocation to estimate the MEAV 95 of ropivacaine 0.75%. Successful ISB was defined as complete absence of pinprick sensation in the C5 and C6 dermatomes 30 min postblock. Secondary outcomes assessed included ability to complete surgery with propofol sedation, change in slow vital capacity, room air oxygen saturation postblock, block duration, ISB complications, and numeric rating scale for pain immediately after surgery.

**Results**: The study was stopped early due to futility. Among 225 participants approached, 54 consented to participate. The MEAV 95 for ultrasound-guided ISB of ropivacaine 0.75% for shoulder surgery was unable to be accurately estimated. Local anesthetic volumes between 5 and 20 ml did not influence any of the predefined secondary outcomes.

**Conclusions**: The MEAV 95 (at 30 min) of ropivacaine 0.75% for ultrasound-guided ISB exceeds the local anesthetic volumes that consistently produces hemidiaphragmatic impairment. ISB cannot be guaranteed to provide surgical anesthesia at 30 min without the potential for concomitant phrenic nerve block.

## RÉSUMÉ

**Contexte**: Le bloc du plexus brachial par approche interscalénique (BIS) est régulièrement utilisé pour l’anesthésie et l’analgésie lors de chirurgies de l’épaule. Les volumes d’anesthésique local traditionnellement utilisés pour le BIS entraînent couramment une parésie ipsilatérale du nerf phrénique compromettant l’oxygénation et la ventilation.

**Objectifs**: Le but de cette étude était de déterminer le plus petit volume minimal effectif chez 95% des patients (VME 95) de la ropivacaïne 0,75 % lorsqu’utilisé pour un BIS lors d’une chirurgie arthroscopique de l’épaule.

**Méthodes**: Étude de cohorte prospective observationnelle auprès de patients subissant une chirurgie arthroscopique de l’épaule sous BIS (niveau C6) avec sédation. Le protocole d’identification du VME 95 de la ropivacaïne 0,75% a été effectué selon la règle de Narayana pour augmenter ou diminuer l’allocation séquentielle des doses. Un BIS réussi était défini comme l’absence totale de sensation de picotement dans les dermatomes aux niveaux C5 et C6 30 minutes après le bloc. Les critères d’évaluation secondaires comprenaient la capacité de compléter la chirurgie avec une sédation à base de propofol, les changements dans la capacité vitale lente, la saturation de l’oxygène de l’air ambiant après le bloc, la durée du bloc, les complications rencontrées avec le BIS et l’intensité de la douleur immédiatement après la chirurgie mesurée à l’aide d’une échelle numérique.

**Résultats**: L’étude a été arrêtée avant terme en raison de son insuccès. Parmi les 225 patients approchés, 54 ont accepté d’y participer. Le VME 95 de la ropivacaïne 0,75 % pour un BIS écho-guidé lors d’une chirurgie de l’épaule n’a pu être estimé avec précision. Les volumes d’anesthésique local entre 5 et 20 ml n’ont influencé aucun des critères d’évaluation secondaires prédéfinis.

**Conclusions**: Le VME 95 de la ropivacaïne 0,75 % (à 30 minutes) pour un BIS écho-guidé excède les volumes d’anesthésique local qui entraînent systématiquement une déficience au niveau du l’hémidiaphragme. Le BIS ne peut fournir une anesthésie de 30 minutes pour une chirurgie arthroscopique de l’épaule sans risque concomitant de bloquer le nerf phrénique.

## Introduction

Interscalene brachial plexus block (ISB) is commonly used for surgery on the upper limb, is regarded as the standard of care for analgesia after shoulder surgery, and reduces opioid consumption.^1,2^ It provides reliable analgesia to the shoulder, lateral aspects of the arm, and the forearm. In the majority of patients, the benefits outweigh the risks by reducing ventilatory depression associated with significant opioid use after painful upper extremity surgery. Shoulder surgery, previously requiring inpatient admission for pain control, is now commonly performed on an ambulatory basis facilitated by ISB analgesia. The most common, clinically significant risk of ISB, however, is near universal ipsilateral phrenic nerve block resulting in diaphragmatic paresis,^3–5^ which results in a 30% reduction in pulmonary function (forced vital capacity and forced expiratory flow rate at 1 s) on average.^6^ It is recommended that ISB be avoided in patients with significantly reduced pulmonary reserve as it can result in clinically significant hypoxia and potentially, prolonged mechanical ventilatory support.^5,7,8^ Unfortunately, this limits the use of ISB in the very population that may benefit the most from reduced opioid consumption and the avoidance of general anesthesia.

The traditional volume of local anesthetic administered for surgical anesthesia is between 20 and 40 ml, which results in proximal spread to the cervical plexus (C3–C4) and anterior spread to the cervical sympathetic chain, recurrent laryngeal nerve, and phrenic nerve.^9^ This results in Horner’s syndrome, hoarseness, and hemidiaphragmatic paresis, respectively, for the duration of local anesthetic action. Ultrasound guidance provides the ability to more accurately target local anesthetic deposition to reduce volumes even further yet maintain analgesic efficacy. Though a reduction in volume for ISB from 20 to 10 ml did not demonstrate an appreciable decrease in the incidence of hemidiaphragmatic paresis,^10^ a reduction to 5 ml in two studies demonstrated 55% and 67% relative reductions in the incidence hemidiaphragmatic paresis.^11,12^ In addition to the reduced incidence of phrenic nerve paresis, its profile is altered in low-dose ISB with shorter duration and a milder impairment in diaphragmatic function evidenced by the markedly superior pulmonary function tests in the low-dose ISB group compared to traditional volumes.^13^ These data suggest that phrenic nerve paresis encompasses a spectrum of severity that varies with dose and volume.

The accuracy of ultrasound guidance has facilitated reduced volumes of local anesthetic for ISB while maintaining effective analgesia with volumes between 1 and 10 ml^10,12,14–17^ Current estimations of the minimal effective anesthetic volume (MEAV) of ropivacaine 0.75% for ISB to provide surgical anesthesia at 95% efficacy (MEAV 95) are suboptimal because they are extrapolated from the minimal effective anesthetic volume for 50% of patients (MEAV 50). Extrapolated values produce unreliable estimates with wide confidence intervals.^18^ The aim of this study was to determine an accurate estimation of the MEAV 95 (at 30 min) of ropivacaine 0.75% for surgical anesthesia in arthroscopic shoulder surgery and whether the estimated volume could preserve diaphragmatic function. Our hypothesis was that the MEAV 95 required to produce surgical anesthesia is lower than the local anesthetic volume used in current practice of 20 to 40 ml.

## Methods

This single-center, prospective, observational study was conducted at Sunnybrook Health Sciences Centre. The Research Ethics Board of Sunnybrook Health Sciences Centre approved this study in October 2012. Adult patients (18 to 75 years of age) with American Society of Anesthesiologists statuses I–III, scheduled to undergo elective arthroscopic shoulder surgery were eligible for enrollment. Written and informed consent was obtained from all participants. Exclusion criteria included patient refusal or inability to provide informed consent, preexisting severe chronic obstructive pulmonary disease (forced expiratory volume in 1 s < 40%), unstable asthma, preexisting diaphragmatic dysfunction, coagulopathy, infection at injection site, allergy to local anesthetic or any other study drug, chronic opioid use (>30 mg oral morphine or equivalent/day), and body mass index > 40.

On the day of surgery, participants had intravenous access established and routine monitors including electrocardiography, noninvasive blood pressure, and pulse oximetry applied. Per institutional multimodal analgesic practice, oral acetaminophen (1000 mg) and celecoxib (400 mg) were administered. Supplemental oxygen (6 L.min^−1^ facemask) and sedation with midazolam 0.03 mg.kg^−1^ were administered. Participants were positioned for a posterior approach to ISB and the skin was cleansed with 70% alcohol/2% chlorhexidine solution. ISB was performed using a 50-mm 22-gauge blunt block needle (B. Braun, Melsungen, Germany) by six regional anesthesia fellowship trained staff anesthesiologists or supervised regional anesthesia fellows with ultrasound guidance (Sonosite M-Turbo, Bothell, WA) and nerve stimulation at the anesthesiologist’s discretion. The needle tip was positioned adjacent to the C5 and C6 nerve roots and ropivacaine 0.75% (predetermined volume per dose finding protocol) was injected to achieve circumferential spread of local anesthetic around the plexus. The anesthesiologist performing the ISB and research assistant conducting postblock assessments were blinded to volume injected. The local anesthetic was prepared by a block room assistant and then covered with opaque tape to blind all other individuals to volume injected. Sensory and motor block function at the C5/C6 dermatomes and the deltoid area were assessed at 10-min intervals for 30 min. Ipsilateral hemidiaphragmatic excursion was assessed by ultrasonography at the cephalad border of the zone of apposition of the diaphragm to the costal margin between the mid-clavicular and anterior axillary lines using a 2- to 5-MHz curvilinear probe (Sonosite M-Turbo). Diaphragmatic movement was assessed in both B-mode and M-mode settings as described by Ayoub and colleagues.^19^ Normal inspiratory caudad excursion was designated as positive motion and paradoxical cephalad motion as negative motion. Bedside spirometry using a compact spirometer (Spirolab III, Medical International Research, Rome, Italy) was performed with patients lying in a semirecumbent position. After instruction on how to perform the test, slow vital capacity (SVC) measurements were performed three times and the values were averaged. Sensation of the upper extremity was assessed by pinprick using a 23-guage needle testing from C4 to T1 dermatomes and scored as full sensation (2), diminished (1), or absent (0). The motor power assessment of the deltoid and biceps was scored as movement present (2), diminished (1), and absent (0). All of the above assessments (diaphragmatic excursion, spirometry, sensory, and motor assessment) were done at baseline (preblock), and 30 min postinjection.

Successful surgical anesthesia was defined by complete absence (score of 0) to pinprick sensation in the C5 and C6 dermatomes at 30 min. Surgery was performed under moderate sedation, with propofol infusion (0.05–0.1 mg.kg^−1^) titrated to the Richmond-Agitation Sedation scale (RASS) of −3 to −4 (Supplemental Appendix A). The arthroscopic port sites were infiltrated with 10 ml 0.5% bupivacaine and 1:200 000 epinephrine prior to incision. In the event of the predefined failure outcome at 30 min, the protocol stipulated that the attending anesthesiologist could still proceed under sedation if deemed feasible. Patients experiencing discomfort despite sedation were converted to general anesthesia with laryngeal mask airway for the remainder of the procedure. Postoperatively, patients were monitored in the recovery room for 2 h or until meeting ambulatory discharge criteria.

### Outcomes assessed

The primary outcome was block success defined as complete absence of pinprick sensation in the C5/C6 dermatomes 30 min after completion of local anesthetic injection. The strict definition of block success was chosen to balance both internal and external validity. Research assistants blinded to injection volume assessed pinprick sensation rather than investigators to minimize bias and increase internal validity. Thirty minutes was selected because it was thought to increase external validity/generalizability and it was felt that most anesthesiologists/surgeons would be unwilling to wait longer than this period to achieve surgical block. Secondary outcomes included ability to complete surgery with propofol sedation (RASS −3/−4), change in SVC (in liters) as a measure of diaphragmatic function 30 min after completion of local anesthetic injection, block duration defined as time from end of injection to first opioid consumption, and Numeric Pain Rating Scale (NPRS) on arrival to the postanesthesia care unit (PACU). Room air oxygen saturation 30 min after completion of local anesthetic injection and complications of ISB (hoarseness, Horner’s syndrome, hypoxia requiring inpatient admission) were also recorded.

### Dose finding protocol

According to the sequential dose finding protocol, each patient’s response determines the volume of ropivacaine 0.75% for the next patient using the up–down design for sequential allocation modified by the Narayana rule to cluster the dose around the effective dose, 95% (ED95). Of the several variations of up–down designs developed, the Narayana rule has been shown in simulations to provide more precise estimations in most cases, compared to the other approaches.^20^

The allocation algorithm is described below:

Suppose the *n*th patient was allocated to level *dj, j* = 1, 2, …, *K*.
*Xj*(*n*) and *Nj*(*n*) are the number of success responses and number of assignments to the dose *dj* up to and including the *n*th patient.
  Assign the next patient to
  1. dose level *dj* − 1 if *Xj*(*n*)/*Nj*(*n*) > 0.95 and if all success responses among the 14 most recent responses on the current dose level.
  dose level *dj* + 1 if *Xj*(*n*)/*Nj*(*n*) < 0.95 and if <14 success response among the 14 most recent responses on the current dose level;
  otherwise, assign to dose level *dj*.

In the event of block failure, the volume for the next patient is increased by 1 ml if the overall success rate at that volume level is <95%. This procedure is repeated until there is a responder—a patient with a complete sensory block at 30 min. The selection of the next dose depends on satisfaction of conditions 1, 2, and 3, based on the number of patient assignments that have taken place and the number of previous successes and failures. This design guarantees that the majority of the measured responses will be in the vicinity of the ED95. The starting dose selected was 5 ml of ropivacaine 0.75%, because this is the lowest dose demonstrating 100% block success for surgical anesthesia in the current literature.^14^ Based on simulation studies of 12 000 scenarios with Python 2.7.3 (with NumPy, SciPy, matplotlib; Python Software Foundation, Beaverton, Oregon, USA), it was determined that 60 participants would be required to make an accurate estimation of the MEAV 95. This design is superior to the more commonly employed Dixon and Mood method where the ED95 is estimated from the calculated ED50. With the Narayana rule applied to standard up–down methodology, the probability of assigning a dose not among that closest to the designated target, in this case ED95, approaches zero.^20^

### Statistical analysis

Data were analyzed with R 3.1 (R Foundation for Statistical Computing, Vienna, Austria; https://www.R-project.org). For the primary outcome of block success, bias-reduced logistic regression using the *logistf* function was performed with ISB volume as the predictor variable. The regression tries to fit a positive slope for ISB volume versus block success using the following formula:

$$\ln\left(\frac{F\left(x\right)}{1-F\left(x\right)}\right)=\alpha +\beta x.$$

*F* is designated as the success level desired (0.95 for MEAV 95) and β is the volume of ropivacaine 0.75%.

Secondary outcomes were analyzed with linear regression (diaphragmatic impairment, block duration) or logistic regression (general anaesthesia versus sedation, NPRS ≥ 3 in PACU).

## Results

Among 225 potential participants, 54 provided written and informed consent. Seventy-nine patients had at least one criterion for exclusion, 86 declined to participate, and six patients were excluded because study staff were unavailable. Demographic characteristics are detailed in Table 1. The specific ISB volume allocated to each participant and the sequence of positive/negative responses are detailed in Table 2. Participants 30 through 34 received an inappropriate ISB volume (Table 2) secondary to transcription error.

**Table 1.** Demographics.^a^

Age	57.0 (10.1)
BMI	29.1 (4.5)
Gender (male/female)	38 [70]/16 [30]
Surgical side Right/Left	31 [57]/23 [43]
ASA I/II/III	13 [24]/30 [56]/11 [20]
Preop NPRS	2.7 (2.4)

^a^Data presented as mean (SD) or count [%]. BMI = body mass index; ASA = American Society of Anesthesiologists’ physical status classification; NPRS = Numeric Pain Rating Scale.

**Table 2.** Sequence of patients and ISB volumes.

Allocation no.	ISB (mL)	Block success^a^	Anesthetic type
1	5	Y	Propofol sedation
2	5	Y	Propofol sedation
3	5	Y	Propofol sedation
4	5	Y	Propofol sedation
5	5	N	General
6	6	Y	Propofol sedation
7	6	Y	Propofol sedation
8	6	Y	Propofol sedation
9	6	Y	Propofol sedation
10	6	Y	Propofol sedation
11	6	Y	Propofol sedation
12	6	Y	Propofol sedation
13	6	Y	Propofol sedation
14	6	Y	Propofol sedation
15	6	Y	Propofol sedation
16	6	Y	Propofol sedation
17	6	Y	Propofol sedation
18	6	Y	Propofol sedation
19	6	N	General
20	7	Y	Propofol sedation
21	7	Y	Propofol sedation
22	7	Y	Propofol sedation
23	7	Y	Propofol sedation
24	7	Y	Propofol sedation
25	7	Y	Propofol sedation
26	7	N	General
27	8	N	Propofol sedation
28	9	Y	Propofol sedation
29	9	N	Propofol sedation
30	9	N	Propofol sedation
31	9	Y	Propofol sedation
32	9	N	General
33	9	N	Propofol sedation
34	9	Y	Propofol sedation
35	10	Y	Propofol sedation
36	10	Y	Propofol sedation
37	10	Y	Propofol sedation
38	10	Y	Propofol sedation
39	10	Y	Propofol sedation
40	10	Y	Propofol sedation
41	10	Y	Propofol sedation
42	10	Y	Propofol sedation
43	10	N	General
44	11	N	Propofol sedation
45	12	N	General
46	13	Y	Propofol sedation
47	13	N	General
48	15	N	Propofol sedation
49	16	N	Propofol sedation
50	17	N	Propofol sedation
51	18	N	Propofol sedation
52	19	N	Propofol sedation
53	20	Y	Propofol sedation
54	20	Y	Propofol sedation

^a^Block success defined as complete absence of pinprick sensation at C5 and C6 dermatomes. ISB = interscalene brachial plexus.

The study was stopped early for futility at 54 participants, although the initial protocol was approved for 60 participants. Based on the Narayana up–down methodology employed, there was no reasonable prospect of the MEAV 95 being less than 10 ml. Secondly, we did not wish to exceed injection of 150 mg of ropivacaine to reduce the chance of local anesthetic systemic toxicity. The ability to achieve complete loss of sensory and motor function in the C5 and C6 dermatomes at 30 min, with 95% reliability, is greater than 10 ml, a volume that has demonstrated 100% diaphragmatic paresis in previous studies.^10,11^ Based on the data, an accurate estimation of the MEAV 95 for block success at 30 min with the logistic regression model was not possible (*P* = 0.592).

ISB volumes between 5 and 20 ml of 0.75% ropivacaine did not influence the ability to complete surgery under light sedation versus general anaesthesia, block duration, incidence of NPRS ≥ 3 in PACU, room air oxygen saturation 30 min postblock, or changes in SVC (Tables 3 and 4). No participant experienced complications associated with ISB.

**Table 3.** Secondary outcomes.^a^

Block success (Y/N)	37 [69]/17 [31]
Sedation/general anesthetic	45 [83]/9 [17]
Block duration (h)	12.8(4.7)
NPRS ≥ 3 in PACU (Y/N)	6 [11]/48 [89]
Room air SpO_2_ 30 min postblock	96.3(2.0)
Mean decrease in SVC (L)	1.1(0.6)
Hemidiaphragmatic paresis (none/partial/complete)	21 [39]/29 [54]/4 [7]
ISB complications (Y/N)	0 [0]/54 [100]

^a^Data presented as mean (SD) or count [%]. NPRS = Numeric Pain Rating Scale; PACU = postanesthesia care unit; SVC = slow vital capacity; ISB = interscalene brachial plexus block.

**Table 4.** Influence of ISB volume.

Outcome	Regression	Predictor variables	*P* value
Anesthetic type	Logistic	Block volume	0.483
Diaphragmatic dysfunction (SVC)	Linear	Block volume, gender, side (right vs. left), BMI	0.061
Block duration	Linear	Block volume	0.312
NPRS in PACU ≥ 3	Logistic	Block volume Preop NPRS	0.915

ISB = interscalene brachial plexus block; SVC = slow vital capacity; BMI = body mass index; NPRS = Numeric Pain Rating Scale; PACU = postanesthesia care unit.

## Conclusions

This prospective, observational study employing the Narayana up–down method attempted to determine the MEAV 95 of ropivacaine 0.75% for ISB to achieve surgical anesthesia at 30 min. We were unable to make an accurate estimation of the MEAV 95. ISB volumes between 5 and 20 ml do not reliably predict complete absence of sensorimotor function in the C5 and C6 dermatomes at 30 min postblock. Despite not achieving the predefined objective outcome of block success at 30 min, the majority of procedures were completed under sedation with minimal to no pain in the recovery room after surgery. However, the absence of diaphragmatic dysfunction and surgical anesthesia cannot be guaranteed at 30 min. The data demonstrate that volumes between 5 and 20 ml do not significantly influence the predefined outcomes measures of this study.

Our results differ from previous work by Gautier and colleagues, who demonstrated that the ISB MEAV 50 of ropivacaine 0.75% for surgical anesthesia at 30 min was 5 ml.^14^ Gautier and colleagues defined block success as any of the following: (1) absence of sensation over the deltoid; (2) unable to abduct/flex arm against resistance (or weaker); or (3) ability to complete surgery under propofol sedation (titrated to Ramsey scale of 5). Possibly contributing to our inability to determine the MEAV 95 was very stringent, though entirely objective, primary outcome definition of block success that stipulated a complete absence of pinprick sensation in the C5/C6 dermatomes at 30 min. This objective outcome was specifically selected to minimize bias. However, the majority of patients in our study (45 of 54) were able to complete surgery under ISB with propofol sedation titrated to a RASS −3 to −4 (less sedation than RSS 5). Furthermore, the number of anesthesiologists (six) performing/supervising the ISB may have introduced heterogeneity to our results and contributed to the contrasting results in the previous literature.

Of the procedures completed under sedation, all demonstrated complete absence of sensorimotor activity in the C5/C6 dermatomes greater than 30 min postinjection that were nonetheless defined as block failure due to time elapsed. We were able to observe this because our institution has a sufficient volume of peripheral nerve blocks to warrant staffing of a separate block room and allow blocks to be completed well in advance of entering the operating theater. This model has limited generalizability to most institutions where nerve blocks are performed in the operating theater and time/efficiency pressures require faster block onset. Consequently, the ability to achieve surgical anesthesia with ISB for shoulder surgery within 30 min and avoid hemidiaphragmatic paresis is challenging.

## Supplementary Material

Appendix_A_G___Richmond_Agitation_Sedation_Scale.pdf
