# Supplementary material for: The minimal effective volume (MEAV 95) for interscalene brachial plexus block for surgical anesthesia under sedation: A prospective observational dose finding study
Source: Can J Pain. 2017 Jun 26;1(1):8–13. doi: 10.1080/24740527.2017.1304805 (PMC8730609; doi:10.1080/24740527.2017.1304805)
Supplement: Appendix_A_G___Richmond_Agitation_Sedation_Scale.pdf [file UCJP_A_1304805_SM4727.pdf]

Appendix A – Richmond Agitation Sedation Scale

Score	Term	Description
+4	Combative	Combative, violent, immediate danger to staff
+3	Very Agitated	Pulls or removes tubes/catheters, aggressive
+2	Agitated	Frequent non-purposeful movement
+1	Restless	Anxious, apprehensive, movements not aggressive
0	Alert and calm	Spontaneous pays attention to caregiver
-1	Drowsy	Not fully alert, but sustained awakening with eye contact to verbal stimulation
-2	Light sedation	Briefly awakens with eye contact to verbal stimulation
-3	Moderate sedation	Any movement (no eye contact) to verbal stimulation
-4	Deep sedation	No response to verbal stimulation, movement to physical stimulation
-5	Unrousable	No response to verbal/physical stimulation
